# Advanced Impedance Spectroscopy for QCM Sensor in Liquid Medium

**DOI:** 10.3390/s22062337

**Published:** 2022-03-17

**Authors:** Ioan Burda

**Affiliations:** Physics Department, Babes-Bolyai University, 400084 Cluj-Napoca, Romania; ioan.burda@ubbcluj.ro

**Keywords:** QCM sensors, in-liquid measurements, virtual instrumentation, impedance analysis, piezoelectric materials

## Abstract

Technological evolution has allowed impedance analysis to become a versatile and efficient method for the precise measurement of the equivalent electrical parameters of the quartz crystal microbalance (QCM). By measuring the dissipation factor, or another equivalent electrical parameter, the QCM sensor provides access to the sample mass per unit area and its physical parameters, thus ensuring a detailed analysis. This paper aims to demonstrate the benefits of advanced impedance spectroscopy concerning the Butterworth–van Dyke (BVD) model for QCM sensors immersed with an electrode in a liquid medium. The support instrument in this study is a fast and accurate software-defined virtual impedance analyzer (VIA) with real-time computing capabilities of the QCM sensor’s electric model. Advanced software methods of self-calibration, real-time compensation, innovative post-compensation, and simultaneous calculation by several methods are the experimental resources of the results presented in this paper. The experimental results validate the theoretical concepts and demonstrate both the capabilities of VIA as an instrument and the significant improvements brought by the advanced software methods of impedance spectroscopy analysis related to the BVD model.

## 1. Introduction

The QCM sensor has been used as a label-free biosensor in recent decades, after its ability to operate in a liquid medium [1,2,3] was demonstrated. The QCM sensor has been adopted as a biosensor because it is versatile and durable, involving both a simple and very stable structure. The QCM sensor has numerous nanoscale applications, including cells [4], antibody interactions [5], detection of DNA hybridizations [6], and viruses [7]. However, the application of piezoelectric materials as biosensors is a challenge due to losses induced by contact with the liquid medium. The detection limit for the QCM sensor is lower in comparison with surface acoustic wave (SAW) sensors [8]. Furthermore, with regard to label-free biosensing in liquids, surface plasmon resonance (SPR) spectroscopy [9] has a limit of detection lower than the QCM sensor. On the other hand, the QCM sensor provides access to the physical parameters of the sample by measuring the dissipation factor or another equivalent electrical parameter, ensuring a detailed analysis of the surface and interactions on the surface without being limited to mass measurement per unit area. Based on the dissipation parameter, the viscoelastic and conformational properties of the sample [10] are monitored. The most powerful methods based on passive interrogation are impedance analysis (QCMI) [11,12] and the ringdown method (QCM-D) [13,14,15]. 

The methods regularly found in the literature focus exclusively on improving the accuracy of resonant frequency and the dissipation factor measurement. This is because resonance frequency and the dissipation factor are very valuable parameters for evaluating the behavior of the QCM sensor in a liquid medium. It is important to note at this time that the measurement of all parameters of the Butterworth–van Dyke (BVD) model ensures a clear picture of the interactions at the surface of the QCM sensor, which is always harshly dependent on experimental circumstances. The use of the QCM sensor in experimental circumstances characterized by temperature and flow gradients is subject to uncertainty. 

Eliminating uncertainty implies an ideal configuration of the experimental setup for the QCM sensor. An ideal configuration, from which both internal and external factors that disturb the stability of the QCM sensor are eliminated, cannot be achieved experimentally because it is impossible to make portable equipment for large-scale applications of the QCM sensor. Temperature changes [16] mostly affect the behavior of the QCM sensor, and active temperature control can in turn become a noise source. If the liquid medium is biological, then the working temperature is imposed by it, and its stabilization usually involves significant experimental resources. The measuring cell, regardless of its topology, exerts mechanical stress on the QCM sensor, having the effect of reducing its quality factor (Q) and finally resulting in the degradation of the frequency stability. Despite all the advances made in the field of electronic devices, their thermal noise negatively influences the stability of the QCM sensor, especially in active measurement configurations. The jitter of the periodic signal used as a clock reference in modern passive measurement instruments is also a disturbing parameter that has the effect of reducing the resolution of the QCM sensor. If the QCM sensor is used in a liquid environment, then the pressure [17] or flow [18,19] differences induced by the pumping system will also have a negative influence on the stability.

The use of external sensors to monitor environmental influences is possible, but the evaluation of real-time corrections is difficult due to the propagation times of these influences in the experimental setup being simultaneous with the changes that take place on the surface of the QCM sensor. An instrument based on impedance analysis that can monitor in real time all the parameters of the BVD model would eliminate a misinterpretation of the external events that occurred during the measurement. In this way, even if the external influences are not eliminated, their identification would always be possible by measuring the impedance of the QCM sensor. With each decade, the cost of impedance analyzers has decreased [20], so the major disadvantage related to their cost has now been eliminated.

Based on the recent concept in instrumentation, a fast and accurate virtual impedance analyzer (VIA) was developed [21]. Today we can consider that a modern VIA must ensure the real-time calculation of the equivalent electrical parameters for the QCM sensor. The concept of a virtual instrument implies the existence of a field programmable gate array circuit (FPGA) in which configurable hardware modules are implemented and analog support is provided by an analog-to-digital converter (ADC) and digital-to-analog converters (DACs).

This paper aims to demonstrate the benefits of advanced impedance spectroscopy relative to the Butterworth–van Dyke (BVD) model for QCM sensors immersed in a liquid medium. The VIA defined by software with real-time computation capabilities for QCM sensor parameters covers a wide range of measurements for the impedance. Advanced software methods, such as self-compensation, post-compensation, and simultaneous computation by using a multimethod based on the BVD model, are the source of experimental and theoretical results presented in this paper. In the literature, few advanced electrical models were proposed [22,23,24,25,26] to solve the limitations of the BVD model in a liquid medium. Some electrical models [27], inspired by the field of impedance spectroscopy, are not related to the BVD model or based on mechanical or acoustic analogy. 

This paper makes the following contributions: (i) advanced QCM sensor characterization based on BVD model in a liquid medium; (ii) valuable consideration of the BVD model in accord with the energetic transfer model; (iii) an innovative post-compensation strategy based on the Nyquist plot; and (iv) a theoretical and experimental study of the capabilities of the BVD model to describe the QCM sensor immersed in a liquid medium. Experimental validation of proposed new post-compensation strategies based on the Nyquist plot was performed in a liquid medium with different viscosities. In this way, the advantages of compensation software techniques for the realization of high-performance applications in the area of QCM sensors are confirmed without resorting to dedicated electronics analog circuits or specialized hardware resources.

## 2. Materials and Methods

### 2.1. Virtual Impedance Analyzer

Traditionally, a simplified “active” method, in which the QCM sensor is part of an oscillator circuit, is frequently used. The amplifier contained in this circuit has a certain influence on the oscillation frequency. For this reason, “passive” methods are more accurate, and even if they are more sophisticated, they are preferred. Of these, the impedance analysis method based on passive interrogation is commonly used in applications [20] to investigate the behavior of the QCM sensor. 

**Figure 1:**
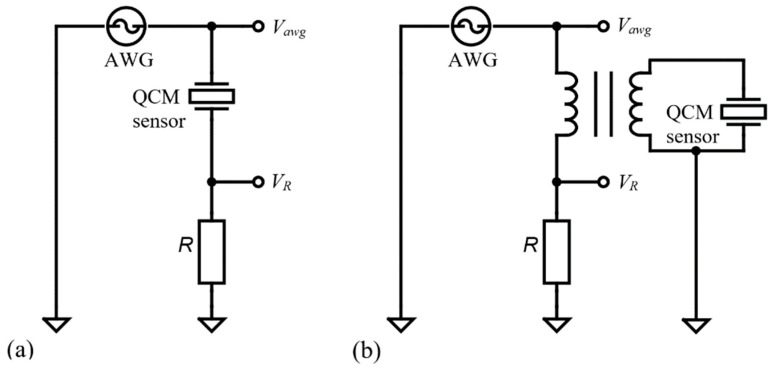
Methods of the impedance analysis. (**a**) Half-bridge configuration for wide dynamic range. (**b**) With one grounded electrode based on a balun transformer.

Figure 1a shows the basic configuration for passively measuring the impedance of a QCM sensor in air. This configuration can provide a wide impedance measurement range. The configuration shown is of the voltage divider or half-bridge type with a passive interrogation in the resonant frequencies range of the QCM sensor. The high impedance analog inputs ensure the measurement, in digital synchronism conditions, of the voltages $V_{awg}\left(\omega \right)$ and $V_{R}\left(\omega \right)$. The impedance of the QCM sensor Z(ω) is calculated, taking into account a reference resistance R. In this case, the impedance of the QCM sensor, in terms of voltage, is given by the relation

(1) $$Z\left(\omega \right)=R\left(\frac{V_{awg}\left(\omega \right)}{V_{R}\left(\omega \right)}-1\right).$$

The configuration shown in Figure 1a is recommended for measuring the impedance of the QCM sensor with a high-quality factor (Q). This situation is encountered in the air or gaseous medium when the QCM sensor is usually functionalized by using a thin layer. The possibility of being able to amplify (or by using another voltage scale) the voltage on the reference resistance against zero background transforms this configuration into the basic method in impedance measurements.

A QCM sensor in the air has a high parallel resonance impedance and must be wired in the configuration shown in Figure 1a. It is recommended that the electrode in contact with the liquid medium be connected to the ground to provide electrochemistry applications. If grounding of the liquid-exposed electrode is important, a balun transformer and appropriate compensations can be used as shown in Figure 1b. This configuration is mandatory for electrochemistry and recommended in the case of a liquid medium in which its dielectric properties can disturb the resonance of the QCM sensor. 

The most important criteria for a virtual instrument, used in monitoring a QCM sensor’s impedance, are the lowest possible power consumption and the smallest possible physical size. The extensive presentation of VIA used in the experimental setup and the standard compensation concepts [21,28,29,30] are available in the literature. 

Analog Discovery 2 (AD2) from Digilent Inc. (Pullman, WA, USA) [31,32,33,34] is the selected virtual instrument for performing a VIA. The choice of AD2 was determined by the performance of the analog input–output channels. The analog channels have two ADC converters and two DACs, each with a resolution of 14 bits at 100 MSPS. The hardware modules required to make the virtual instrument have been implemented in a Xilinx Spartan 6 FPGA (XC6SLX16-1L). The user has two high-impedance input channels (1 MΩ with 24 pF in parallel) with voltage ranges up to ±25 V. For a voltage range ≤ ±5 V, the absolute resolution is 0.32 mV. The reading of the analog input channels is digitally synchronized, which allows the accurate determination of the phase difference between the input signals. The two analog outputs are typically used by the arbitrary waveform generator (AWG) which provides output signals in the range of ±5 V.

**Figure 2:**
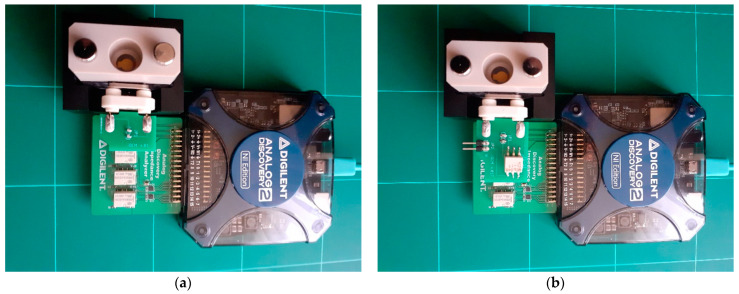
QCM versions based on virtual impedance analyzer. (**a**) Half-bridge configuration for wide dynamic range. (**b**) With one grounded electrode based on a balun transformer.

The hardware interface with the QCM sensor is provided by the shield for the impedance analyzer application [28,29], transforming AD2 into two versions of VIA, as shown in Figure 2. In both versions, an analog-output channel provides the sinusoidal signal of passive interrogation. The AWG interrogation sine wave is applied to the QCM sensor, and its response is measured in digital synchronization conditions through the two analog inputs. The measurement takes place directly on the QCM sensor or with a balun transformer.

The selection of the reference resistance is done with relays, controlled by the digital outputs of AD2. Both topologies shown in Figure 1 have been implemented, as can be seen in Figure 2. The experimental support thus created allows a comparative study about the performance of the advanced software-compensation methods. 

For the topology shown in Figure 2b, the compensation is implicit and without a standard compensation. It is practically impossible to measure the electrical parameters of the BVD model. The experimental setup is completed by the QCM flow cell kit (011121, ALS Co., Ltd., Tokyo, Japan), mounted in its static measurement mode [35].

The PC-type host computer provides the configuration commands, data transfer, and power supply through the USB2 interface of the AD2 virtual instrument. Data acquisition and processing are performed by a Python module that exploits the software development kit (SDK) functions of AD2. The Python module provides real-time processing of experimental raw data and calculates the raw parameters of the BVD model simultaneously by several methods. The acquired raw experimental data are graphically represented together with the parameters of the BVD model. To ensure advanced post-processing in the MATLAB^®^ environment, the raw experimental data are recorded in * .csv files.

### 2.2. The QCM Sensor and BVD Model

**Figure 3:**
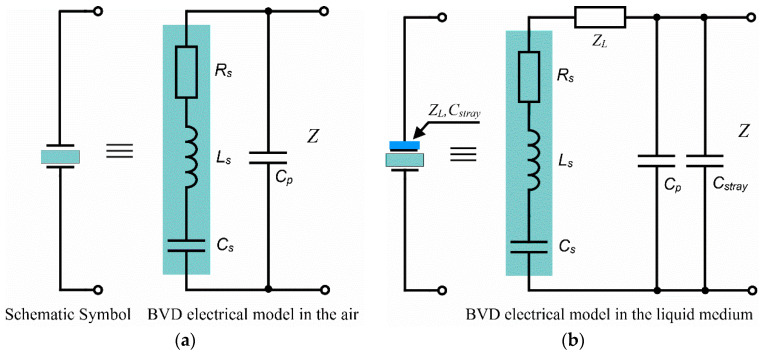
Butterworth–van Dyke (BVD) model of the QCM sensor. (**a**) In the air. (**b**) In the liquid medium.

Based on the energy transfer model, the QCM sensor generates and stores acoustic energy. For this reason, we can consider an analogy with an electrical model consisting of an RCL series circuit to describe its behavior. If the QCM sensor is transformed into a functional sensor, it is covered with material, e.g., a sensitive film. A small part of the acoustic energy is transferred to this sensitive film, which has partly elastic and partly viscoelastic properties. This energy is stored in its elastic part and dissipated in its viscoelastic part. In an ideal approximation of an infinite-quality Q factor of the QCM sensor, the applied electrical power is equal to the sound power at the resonant frequency. Taking this into account, the acoustical impedance is analogous to the electrical impedance. Furthermore, in this process the liquid medium, in which the QCM sensor is used, intervenes. It interacts with the respective materials that are attached to the sensitive film. For the attached materials we must perform an identical consideration about their elastic and viscoelastic properties. Moreover, the dielectric properties of these materials can induce unexpected behavior in the QCM sensor that is reflected in the modification of the electrical parameters. The BVD-lumped electrical model [11] describes the modes of interest of the QCM sensor by using 4 parameters, as shown in Figure 3. 

The arm consisting of the series combination of a resistance $R_{s}$, inductance $L_{s}$, and a capacitance $C_{s}$ is responsible for modeling the storage and dissipation of acoustic energy. Parallel to this arm is a shunt capacitance determined by the existence of electrodes on the two sides of the quartz crystal. The *Z*(*ω*) impedance of the QCM sensor based on the BVD model is determined by the series $Z_{s}\left(\omega \right)$ and parallel $Z_{p}\left(\omega \right)$ impedance combination of the two arms:

(2) $$Z\left(\omega \right)=\frac{Z_{p}\left(\omega \right)Z_{s}\left(\omega \right)}{Z_{p}\left(\omega \right)+Z_{s}\left(\omega \right)}.$$

The reactance of the shunt capacitance $C_{p}$ is given by

(3) $$Z_{p}\left(\omega \right)=-j\frac{1}{\omega C_{p}},$$

and the impedance of the series arm is given by the following equation

(4) $$Z_{s}\left(\omega \right)=R_{s}+j\left(\omega L_{s}-\frac{1}{\omega C_{s}}\right)$$

where $R_{s}$, $L_{s}$, and $C_{s}$ are the parameter of the series arm of the BVD model. 

The BVD model for the QCM sensor with a single electrode in contact with the liquid medium is composed of the impedance of the load $Z_{L}=R_{sL}+j\omega L_{sL}$ in the series arm and a stray capacitance $C_{stray}$ in the parallel arm. The experimental measured raw data for the QCM sensor in the liquid medium is expected to fit with BVD model parameters calculated considering the following equation: $R_{rm}=R_{s}+R_{sL}$, $L_{rm}=L_{s}+L_{sL}$, and $C_{rm}=C_{s}$, where $R_{rm}$, $L_{rm}$, and $C_{rm}$ are the parameter calculated from experimental measurements

(5) $$Z_{s}\left(\omega \right)=\left(R_{s}+R_{sL}\right)+j\left(\omega \left(L_{s}+L_{sL}\right)-\frac{1}{\omega C_{s}}\right).$$

The constraint for the motional capacitance to be constant and equal to its calculated value for the undisturbed state is established by the mechanical or acoustic analogy. The equivalent BVD model derived from theoretical approximations fits the experimental data for the QCM sensor used in the liquid medium. Equation (5) is the key objective of all active or passive methods of measuring the parameters of the QCM sensor. To achieve this objective, many analog topologies [3] and many calibrations and compensation methods have been proposed [36].

In the literature, more models are presented, not relatable by a mechanical or acoustic analogy, inspired by the field of impedance spectroscopy [27]. The analogy of the electrical model to the acoustic load impedance generated by the interaction of the QCM sensor with the liquid medium is mandatory. Any proposed electrical model must be derived from a mechanical or acoustic analogy to be considered as a realistic approach. Experimental investigations have shown a complex behavior for the QCM sensor, such as increasing the resonance frequency [37] under a silicon oil load. In general, for piezoelectric material coupled with other materials, we observe a severe modification in equivalent electrical parameters.

To validate the software advanced impedance analysis based on the BVD model, very precise experimental data obtained by scanning with a small step is used. Another key parameter strongly affected for QCM sensor in liquid medium is the quality factor, Q. The quality factor is related to the half-power spectrum at the resonance frequency. This is also known in terms of the network analyzer as the −3 dB points because the response is down from the maximum by three decibels. In the ringdown measured method [13,14,15], the dissipation factor (D) is preferred to describe the natural acoustic energy losses at the resonance, and it is very simply related to the quality factor by D=1/Q.

### 2.3. Computation of the QCM Sensor Parameters from Raw Experimental Data

#### 2.3.1. Based on the Experimental Quality Factor

The value of the $R_{s}$ series resistance and the $\omega_{r}$ series frequency is determined after considering the sequence of the raw experimental data of the impedance measurements, in the frequency range of the resonant frequencies of the QCM sensor with the help of the minimum search function. These are key parameters of the BVD model, and many instruments are limited to measuring them. The bandwidth Δω is calculated from the intersection of the raw experimental data with a line parallel to the abscissa at $\sqrt{2}R_{s}$. The anti-resonance frequency $\omega_{ar}$ is determined by a maximum search function in the sequence of raw experimental data. Based on the quality factor equation

(6) $$Q=\frac{1}{\omega_{r}R_{s}C_{s}}=\frac{\omega_{r}L_{s}}{R_{s}},$$

QCM sensor parameters that do not result directly from the raw experimental data can be calculated based on the following equations:

(7) $$C_{s}=\frac{1}{\omega_{r}R_{s}Q},$$

(8) $$L_{s}=\frac{QR_{s}}{\omega_{r}},\;\mathrm{and}$$

(9) $$C_{p}=\frac{C_{s}}{{\left(\frac{\omega_{ar}}{\omega_{r}}\right)}^{2}-1}$$

The accuracy of the BVD parameters calculated from the measured raw data depends on the frequency-swiping step as well as on the calibration and compensation procedures. This computation method shown above cannot be applied only after the end of the acquisition process. In other words, the method based on quality factor cannot be used for real-time compensation of the shunt capacitance $C_{p}$.

#### 2.3.2. Based on the Experimental Resonance Frequencies

A very efficient method is based on the direct measurement from the impedance raw experimental data of the series resonance frequency $\omega_{r}$ and the anti-resonance frequency $\omega_{ar}$ with the help of minimum and maximum search functions. The determination of the series resistance $R_{s}$ is implicit in this case. A measurement of the impedance of the QCM sensor at the frequency $\omega_{m}$, less than or higher away from the resonant frequencies where its reactance $Z_{pm}=Z_{p}+Z_{stray}\;$ is purely capacitive, allows the calculation of the shunt and stray capacitance $C_{pm}$ based on the equation

(10) $$C_{pm}=-j\frac{1}{\omega_{m}Z_{pm}}$$

The calculation of the parameters that cannot be determined directly from the raw experimental data is done based on the following equations:

(11) $$C_{s}=C_{pm}\left({\left(\frac{\omega_{ar}}{\omega_{r}}\right)}^{2}-1\right)$$

and

(12) $$L_{s}=\frac{1}{\omega_{r}^{2}C_{s}}$$

Because the shunt and stray capacitance is known at the beginning of the process of measuring the impedance of the QCM sensor, in the area of resonance frequencies, a real-time compensation of the sensor can be obtained.

### 2.4. Standard Method for Impedance Compensation

Modern advanced instruments with data-processing capabilities ensure automatic calibration or self-calibration procedures. This is undoubtedly the main advantage of digital technology for the production of high-precision instruments with extensive data-communication facilities through high-speed interfaces. An advanced impedance analyzer measures the electrical impedance of the QCM sensor around the resonant frequencies. The measurement process is followed by a complete characterization of its response by calculating the electrical parameters of the BVD model. Moreover, if the impedance analyzer is used to identify the parameters of the BVD model for the QCM sensor in a liquid medium, the compensation procedures are of the utmost importance. A minimum and necessary standard compensation is described below.

**Figure 4:**
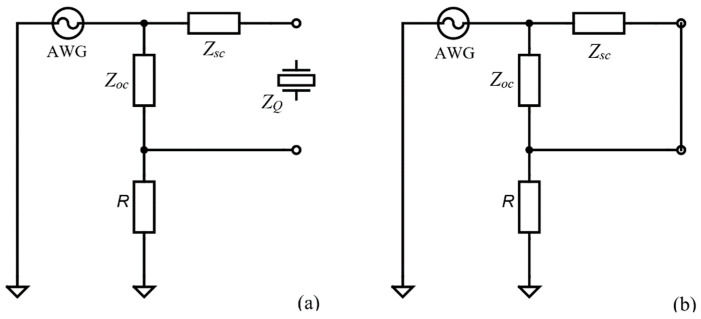
Standard method for impedance compensation. (**a**) Open-circuit compensation. (**b**) Short-circuit compensation.

The standard compensation procedure [30] is shown in Figure 4, which involves the following processes: (i) open-circuit parasitic impedance compensation, $Z_{oc}$ (Figure 4a) followed by (ii) short-circuit parasitic impedance compensation, $Z_{sc}$ (Figure 4b). After $Z_{oc}$ and $Z_{sc}$ have been measured, the actual impedance of the QCM sensor is calculated by using the following equation:

(13) $$Z_{Q}=\frac{Z_{rm}-Z_{sc}}{1-\left(Z_{rm}-Z_{sc}\right)/Z_{oc}},$$

where $Z_{rm}$ is the QCM sensor raw measured impedance.

### 2.5. Real-Time Shunt and Stray Capacitance Compensation

Real-time analog compensation of shunt and stray capacitance is a common topic in the literature [3], and active or passive circuits are used for this purpose. The digital method proposed here is based on Equation (2), and the method of calculating the parameters of the BVD model is presented in Section 2.3.2. Equation (2) can be rewritten in the following form:

(14) $$Z_{rm}=\frac{Z_{s}Z_{p}}{Z_{s}+Z_{p}},$$

where the $Z_{rm}$ is the measured raw impedance. From Equation (14) we can calculate the raw experimental data of the series arm (motional arm):

(15) $$Z_{s}=\frac{Z_{rm}Z_{pm}}{Z_{pm}-Z_{rm}},$$

where $Z_{s}\;\mathrm{is}\;$computed in every measurement iteration based on previous measurements of the $Z_{pm}=Z_{p}+Z_{stray},\;$as was presented in Equation (10). 

The most important advancement of the VIA is related to the capabilities of real-time data manipulation, thus ensuring the functions of compensation through software virtualization and the component of front-end electronics is kept to a minimum. The advantages of software-based instruments in order to customize the hardware of virtual instruments of general use are not only reduced to an economy of electronic devices through the dematerialization of applications, but also through advanced mathematical methods that generate a new level of performance. It is extremely beneficial to replace electronic devices or complex analog circuits with a few program lines. In addition, thermal noise or other parasitic effects specific to analog compensation methods are eliminated.

### 2.6. Advanced Residual Capacitance Compensation in VIA

A natural question about modern virtual instruments based on software and intensive data processing is how far they can go with compensation and self-calibration methods. The only true answer is that technologically we can go up to the physical limit of the investigated phenomena. This situation becomes practically evident when virtual instruments capable of performing complex calculations of a large volume of acquired experimental data are involved. If these calculations are performed in real time, then the measurement and interpretation of the results of measurement becomes a single operation with huge benefits in practice. 

**Figure 5:**
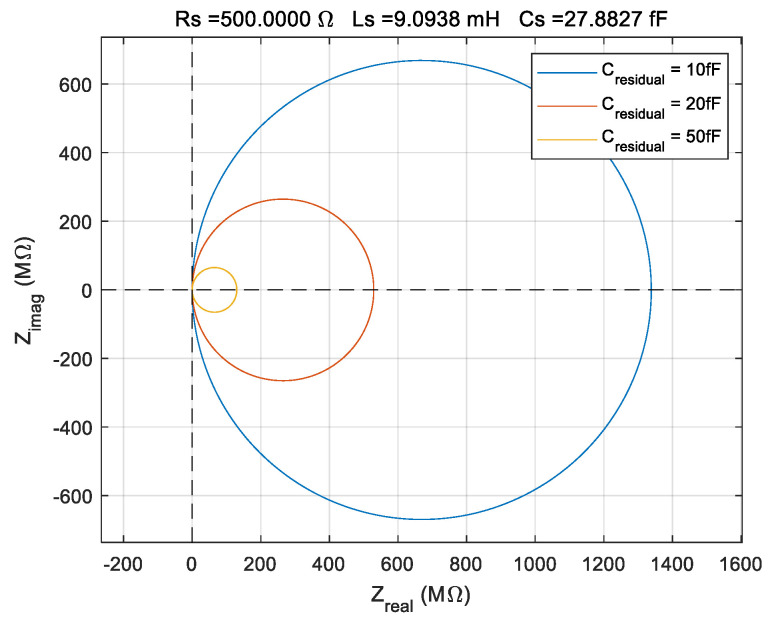
Simulation of the residual shunt and stray capacitance effect as Nyquist plot considering the liquid medium.

Commonly in the literature [3] related to the QCM sensor, QCM sensor frequency response is shown as a Bode plot. This representation of the frequency response of the QCM sensor is very useful in practice but at the same time limited in being able to depict the complexity of the response. The Nyquist Plot or complex-impedance plane representation, in which the data from each frequency point is plotted by the imaginary part on the ordinate and the real part on the abscissa, is very sensitive to changes. It is a common convention in the electrochemistry community to plot $Z_{imag}$ on the y-axis. After the standard compensation procedure described in Section 2.5, the residual capacitance is generated by the imprecision of shunt and stray capacitance measurement. The simulated effect of the residual shunt and stray capacitance is shown in Figure 5.

The residual shunt and stray capacitance $C_{residual}$ are defined by the following equation:

(16) $$C_{residual}=\left|C_{true}-C_{pm}\right|.$$

The simulation from Figure 5 is based on the BVD model considering the values of the parameters typical for a QCM sensor with 10 MHz serial resonant frequency and one electrode immersed in a liquid medium. This residual shunt and stray capacitance are inherently due to the uncertainty of the $C_{pm}$ measurement and perturbations from the experimental environment during raw data acquisition. The Nyquist plot based on the BVD model is made to identify the contribution of the residual capacitance. For $C_{residual}\to 0$, the diameter of the circle in the Nyquist plot is infinite for measurements in an infinite frequency range. In practice, the impedance of the QCM sensor is measured in a narrow range around resonant frequencies. In this situation, the condition for perfect compensation for the shunt and stray capacitance of the QCM sensor is

(17) $$\min\left(Z_{real}\right)-\max\left(Z_{real}\right)\to 0,$$

or in other words, we must have a straight line parallel to the imaginary axis in the Nyquist plot. To minimize residual capacity, an algorithm of successive approximations is used. Because the experimental raw data have a statistical dispersion and are subject to uncertainty, the residual capacity cannot be reduced to zero. In an extremely short post-processing time, the real-time compensated experimental raw data for shunt and stray capacitance is refined to reduce the residual capacitance to less than ±0.1 fF. 

In this new approach specific to virtual instruments, we can make very precise measurements based both on the physical qualities of the electronic devices used and on the computing performance involved in the process. This beneficial change from the perspective of the costs of achieving the experimental setup ensures a new level of performance inaccessible to traditional methods. The self-calibration procedures followed by compensation or adaptive compensation are applicable in different stages: (i) before the data acquisition, (ii) during the data acquisition, or (iii) after the data acquisition based on algorithms usually implemented in the host computer. The ultimate goal of these calibration, compensation, and post-compensation procedures is to obtain a set of experimental data to satisfy Equation (5), the dream of many analog methods. This approach ensures better efficiency of the measurement process than the involvement of methods based on nonlinear fits. The nonlinear fit involves a long calculation time and the final result depends on the initial conditions which in turn are extracted from the raw experimental data.

In conclusion, the Nyquist plot is valuable for identifying how many characteristic features are exhibited by the QCM sensor, and all frequency information is inherently lost. Even if the Nyquist plot is a representation of the same experimental data, or the result of a simulation based on the BVD model, the depiction of the imaginary and real component using two linear axes changes the perspective in impedance analyses.

## 3. Results

In this section, a comparative analysis is performed to validate the proposed software procedures for impedance compensation, shunt and stray capacitance compensation, and advanced residual capacitance compensation relative to the BVD model for the QCM sensor in a liquid medium. The experimental setup is shown in Figure 1 and also contains a QCM sensor made of a quartz crystal with a fundamental resonant frequency of 10 MHz (151225-10, International Crystal Manufacturing Co., Inc., Oklahoma City, OK, USA). The QCM sensor was fixed between the silicon O-rings of the static QCM cell as shown in Figure 1. During the measurements, the temperature in the laboratory was in the range of 21 ± 2 °C, with a relative humidity of 50 ± 10%. The VIA configuration used to obtain the raw experimental data was as follows: (i) passive excitation with a sinusoidal voltage with an amplitude of 1 V in the range of resonant frequencies, and (ii) measurement of the impedance of the QCM sensor at 50,001 points with a swiping step of 1 Hz.

### 3.1. Compensation of the Balun Transformer

The most difficult task for a VIA is to measure the impedance in a large range as is the case when the QCM sensor is in the air. It is recommended that the electrode in contact with the liquid medium be connected to the ground to provide electrochemistry. If grounding of the liquid-exposed electrode is important, a balun transformer and appropriate compensations can be used as shown in Figure 1b. This configuration is mandatory for electrochemistry and recommended in the case of a liquid medium in which its dielectric properties can disturb the resonance of the QCM sensor. A common compensation procedure is shown in Figure 4 and for the balun transformer, the following steps are used: (i) open-circuit compensation to compensate for the open-circuit stray impedance $Z_{oc}$ (Figure 4a), and (ii) short-circuit compensation to compensate for the short-circuit stray impedance $Z_{sc}$ (Figure 4b). Finally, four parameters are used to compensate for the balun transformer: open resistance, open reactance, short-circuit resistance and short-circuit reactance to be consistent with SDK’s compensation function. This parameter must be measured in the resonant frequencies range of the QCM sensor. Assuming the narrow range of the sweeping frequency, the compensation parameters are reduced to four experimental constants. The SDK functions of the AD2 virtual instrument are used to implement the compensation procedures in a single row of the Python module. The radiofrequency transformer T1-1T-KK81 from Mini-Circuits Inc. (Brooklyn, NY, USA) [38] is used as a balun transformer that can ensure a wideband from 80 kHz to 200 MHz with very low return loss. For the open circuit, the resistance and reactance compensation value are 1.11 KΩ and 131.3 Ω respectively. The short-circuit compensation parameters for resistance and reactance are 1.516 Ω and 7.441 Ω, respectively.

**Figure 6:**
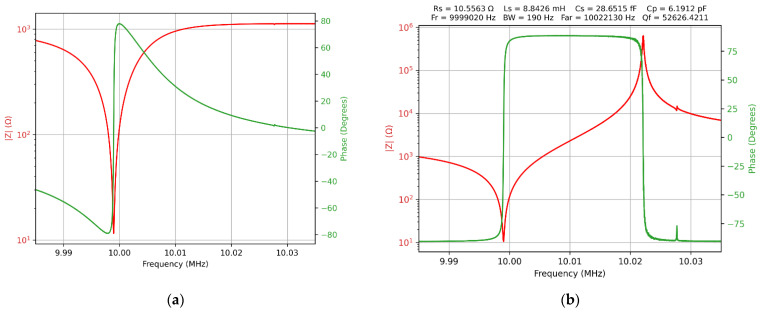
Bode plot of the raw data for a QCM sensor in the air with balun transformer topology. (**a**) Without compensation. (**b**) With compensation.

The effect of compensation for balun-transformer topology is shown in Figure 6. The difference between raw data, shown without compensation in Figure 6a, and raw data with compensation, shown in Figure 6b, is remarkable and confirms the capability of the software-compensation procedures. The spurious resonance [39] present after the anti-resonance peak is highlighted in Figure 6b after the impedance-compensation procedure, and its presence proves the usefulness of software-compensation methods. The experimental configuration evaluated in this section paves the way for the QCM with impedance analysis based on VIA in electrochemistry applications.

### 3.2. Shunt and Stary Capacitance Compensation 

Procedures for the validation of the QCM sensor in high-viscosity liquid medium by using solutions with different glycerin–water concentrations [40] are considered a standard in the evaluation of newly proposed methods. For this purpose, four samples based on deionized water and glycerin were used for the experimental validation of the proposed methods. 

**Figure 7:**
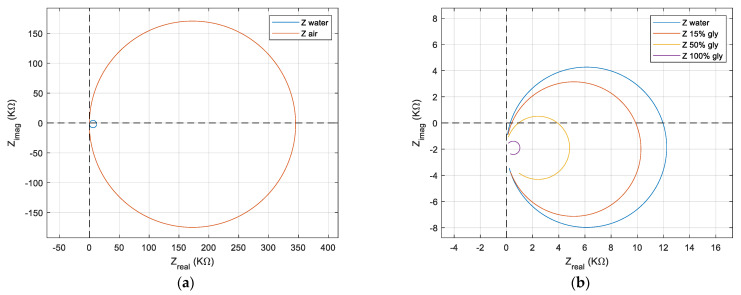
Nyquist plot for QCM sensor. (**a**) In water and air. (**b**) In water, in two concentrations of the glycerin-water solution, and in glycerin.

The significant increase in the total resistance of the motional arm, compared to the minimal change in inductance, negatively affects the quality factor ( Q), which represents the ratio of stored energy to the energy lost in each oscillation period of the QCM sensor. The behavior is mainly influenced by the parallel combination $R_{s}||C_{p}$ close to resonance, which is no longer dominated by the series combination for liquid medium and considerably affects the absolute maximum or minimum impedance points, as well as their phase. Taking advantage of the Nyquist plot, the evolution of the real and imaginary impedance for the QCM sensor induced by the air and liquid medium is shown in Figure 7a. The motional resistance is related to dissipation processes, and this information is contaminated by the existence of shunt and stray capacitance. 

Figure 7a suggestively illustrates the loss of QCM sensor performance in the water. Even with this profound transformation generated by the exposure of an electrode to the liquid environment, the QCM sensor is still a performer. The use of a QCM sensor in the biological liquid medium as a biosensor is a basic application, being a candidate for the implementation of the concept of lab-on-chip. 

The effect generated, in a liquid medium, by the increase in the viscosity is shown in Figure 7b. To illustrate the effect produced by the increase in viscosity in the liquid medium, four samples were taken. The first sample was deionized water followed by two solutions with a concentration of 15% and 50% glycerin in water. A final sample consisting of pure glycerin was used to test at the limit the performances of the proposed compensation software methods. 

As a result, the resonance points in the series and parallel will undergo a translation compared to the case of the QCM sensor in the air where both resonant frequencies occurred around the zero-imaginary crossing point. Based on the Nyquist plot in Figure 7b, the effect of uncompensated shunt and stray capacitance is illustrated with high accuracy. In a liquid medium with increased viscosity, the circles in the impedance locus are shifted in the negative region of an imaginary axis with $\omega_{r}C_{pm}$. For the QCM sensor in the liquid medium, the inductive transition can no longer fully compensate for the capacitive reactance that it has before and after the resonance frequencies. Because shunt and stray capacity have negative effects on the resonant frequency measured in viscous environments, it requires a thorough assessment or compensation for it to eliminate the induced effects. 

The compensation method described in Section 2.5 may be applied in real-time if the shunt and stray capacitance is measured before acquiring data around the resonance frequencies of the QCM sensor. The preliminary determination of the value for the shunt and stray capacitance is made at a frequency far from the resonance frequencies of the QCM sensor in the experiments presented in this paper. This measurement is made at 1 MHz. This measurement of the shunt and stray capacitance cannot be made accurately enough to eliminate any influence in the case of measurements made in high-viscosity liquids.

**Figure 8:**
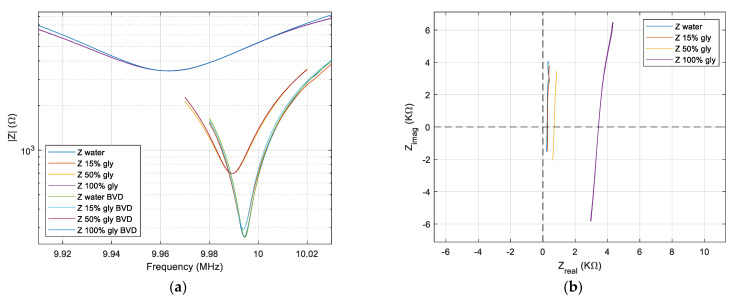
QCM sensor in water, in two concentrations of the glycerin-water solution, and in glycerin after real-time shunt and stray capacitance compensation. (**a**) Bode plot. (**b**) Nyquist plot.

The elimination of the shunt and stray capacitance is possible in real time through the compensation method presented in Section 2.5, and the results are shown in the Bode plot from Figure 8a.

The uncertainty measurements associated with shunt and stray capacitance are the source of the residual capacitance. The presence of the residual capacitance is illustrated in Figure 8b, where the lines are not parallel with the imaginary axis to agree with theoretical expectations described in Section 2.6. If we consider an infinite frequency range, these lines are circles of infinite diameter, as shown in Figure 5, where the existence of very small residual capacitances in the femtofarads range was considered to simulate this effect.

### 3.3. Advanced Compensation of the Residual Capacitance 

By the simulation in Figure 5, the usefulness of the Nyquist plot is evident to identify the existence of a residual parallel capacitance, as yet uncompensated. Advanced compensation of residual capacitance cannot be done in real time. This is a method of post-processing of raw data to eliminate the resulting residual capacitance after real-time compensation of shunt and stray capacitance. In other words, the proposed method decreases the uncertainty of measuring shunt and stray capacitance. As a rule, measuring capacitances with high precision in the case of a non-specialized experimental setup is impossible. 

The QCM sensor is used in non-specialized applications and laboratories in high-precision measurements of electrical parameters, such as in the fields of biology and electrochemistry. Through this advanced method of residual capacitance compensation, this shortcoming is removed. A mathematical compensation criterion, Equation (17), can be used, which results directly from the Nyquist plot. The successive application of two compensation methods, one in real time based on the direct measurement of shunt and stray capacitance, followed by a second advanced compensation, transforms the raw experimental data perfectly fitted with the BVD model. The effect of a residual capacitance, as well as the new advanced compensation method, has been described in extension in Section 2.6. 

**Figure 9:**
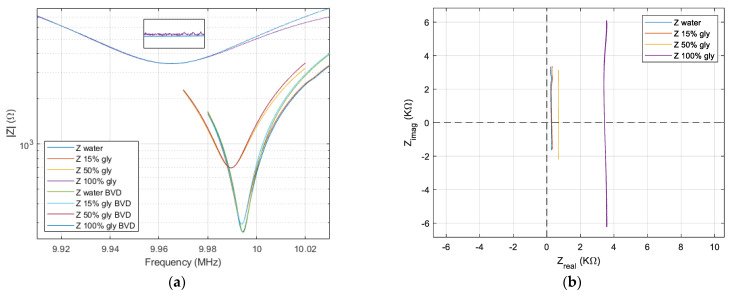
QCM sensor in water, in two concentrations of the glycerin-water solution, and in glycerin after advanced shunt and stray capacitance compensation. (**a**) Bode plot. (**b**) Nyquist plot.

A successive approximation algorithm is used to reduce the residual capacitance to less than ±0.1 fF. The effect of advanced compensation is shown in Figure 9b. In this new situation, the lines are parallel with the imaginary axis in the limit of the statistic distribution of the raw data. Figure 9a shows the raw experimental data after successive compensation presented above fitted on the basis of the Equation (5) by using the raw calculated parameters. A magnification of the fitting around the series resonant frequency is also shown in Figure 9a. 

The maximum measurement error for shunt and stray capacitance is less than 10%. For example, if the operating environment of the QCM sensor is pure glycerin, we have an absolute difference equal to 0.2472 pF, as is shown in Table 1. 

However, due to the high value of motional resistance, the effect of non-alignment of the experimental raw data with the imaginary axis is pronounced, as seen in Figure 8b. By an advanced post-compensation based on procedures presented in Section 2.6, this residual capacitance is practically removed, as can be seen in Figure 9b. The true values for the shunt and stray capacitance are shown in Table 1. 

The BVD model now fits perfectly with the raw experimental data around the series resonance frequency as is shown in Figure 9a. This result is obtained after a double compensation of the shunt and stray capacitance. The parameters of the BVD model can now be read directly from the raw data after a real-time compensation during data acquisition followed by an advanced post-compensation. This procedure is confirmed even if the liquid medium is pure glycerin. The raw data measured in water, 15% glycerol-water solution, 50% glycerol-water solution, and glycerol confirm the expectation about VIA capabilities to manage the impedance measurements of the QCM sensor immersed in a liquid medium. The quality of the impedance measured with VIA for the QCM sensor in glycerin is outstanding. 

## 4. Discussion

Electrical systems, mechanical systems, and acoustical systems are three physically different systems. Frequently, it is sensible to describe some physical systems in terms of electrical analogies. This analogic approach can be considered the birth certificate for many innovative scientific fields such as mechatronics or piezotronics, to name just two. The different physical systems are analogous to each other if the differential equation modeling of these systems is identical. The direct analogy between resistance in the BVD model and acoustic energy dissipation (acoustic resistance), for the QCM sensor, is very useful in the right interpretation of the experimental results. The frequency shift of the resonant frequency $\omega_{r}\left(L_{s}+L_{sL}\right)=1/(\omega_{r}C_{s})$ is correlated with a mass attached to the sensor surface. Both acoustic circuit elements and electrical equivalent elements in a complex conjugate form are responsible for storing energy and are accountable for the resonance frequency. The real source of frequency shift is a modification of the acoustic load impedance, and in this situation, the best fitting is ensured by the best equivalent electrical impedance composed by resistance, inductance, and capacitance. The frequency shift is related to a modification in the elements of the system in charge of energy storage. Acoustical resistance, or in analogy, electrical resistance, reflects all dissipation processes from the QCM sensor, functional layers, and its contact with other materials in a liquid medium. The BVD model is capable of correctly describing the modification in electrical impedance for the QCM sensor around resonant frequencies in a high-viscosity liquid medium after a full compensation of shunt and stray capacitance. The theoretical approach based on the Nyquist plot was experimentally validated in the very harsh medium for the QCM sensor, pure glycerin.

## 5. Conclusions

In this paper, the theoretical and experimental results of compensation and post-compensation strategies in VIA are presented to confirm that it is a very high-performing instrument. In conclusion, the BVD model is capable of correctly describing the modification in electrical impedance for a QCM sensor with an electrode in a liquid medium if we reduce the stray and shunt capacitance to less than 0.1 fF, and small imperfections are generated by statistical dispersion of the experimental raw data. This conclusion is supported from a theoretical point of view and validated experimentally in sections of the paper. Also, the VIA ability to very accurately measure with high resolution the impedance of the QCM sensor in a liquid medium were demonstrated. The virtual impedance analyzer followed by advanced software impedance analysis is the best investigation that we can offer to obtain a sharp picture of the interaction at the surface of the QCM sensor. Moreover, even if the Bode and Nyquist plots reflect the same information only used together, we can create a suggestive and realistic image about the electrical parameters of the QCM sensor relative to the BVD model. Software-defined virtual instruments open multimethod capabilities at this stage, and future versions with advanced reconfigurable front-end electronics topologies are under investigation. These capabilities can be coupled with artificial intelligence (AI) and the Internet of Things (IoT) as a new resource in scientific investigations or environmental monitoring.

## Figures and Tables

**Table 1:** Shunt and stray capacitance and residual capacitance before post-compensation.

	$C_{pm}\;\left(pF\right)$	$\approx C_{true}\;\left(pF\right)$	$C_{residual}\;\left(pF\right)$	%
water	8.5677	9.4331	0.8654	9.17
15% glycerin–water	8.9019	9.4449	0.543	5.74
50% glycerin–water	8.2939	8.7667	0.4728	5.39
100% glycerin	8.2412	8.4884	0.2472	2.91

## Data Availability

Not applicable.
